# Real-Time Antecedents of Young Adults’ Vaping and Co-Vaping of Nicotine and Cannabis: An Ecological Momentary Assessment Study

**DOI:** 10.2196/75695

**Published:** 2025-08-14

**Authors:** Vuong Van Do, Pamela May Ling, Salomeh Keyhani, Gregory M Marcus, Johannes Thrul, Nhung Nguyen

**Affiliations:** 1 Center for Tobacco Control Research and Education, University of California, San Francisco, 530 Parnassus Avenue Suite 366, San Francisco, CA, 94143, United States, 1 415-476-2265; 2 Department of Medicine, Division of General Internal Medicine, University of California, San Francisco, San Francisco, CA, United States; 3 Center for Data to Discovery and Delivery Innovation (3DI), San Francisco VA Health Care System, San Francisco, CA, United States; 4 Department of Medicine, Division of Cardiology, University of California, San Francisco, San Francisco, CA, United States; 5 Department of Mental Health, Bloomberg School of Public Health, Johns Hopkins University, Baltimore, MD, United States; 6 Sidney Kimmel Comprehensive Cancer Center, John Hopkins University, Baltimore, MD, United States; 7 Centre for Alcohol Policy Research, La Trobe University, Melbourne, Australia

**Keywords:** nicotine vaping, cannabis vaping, marijuana vaping, tobacco, co-use, mHealth, mobile phone

## Abstract

**Background:**

Nicotine and cannabis vaping are common among young adults and can potentially lead to adverse health consequences. Identifying real-time antecedents of vaping events may provide insights into intervention targets pertinent to these behaviors. This study aimed to examine real-time antecedents of nicotine and cannabis vaping and same-occasion co-vaping among young adults.

**Objective:**

This study aims to examine real-time antecedents of nicotine and cannabis vaping and same-occasion co-vaping among young adults.

**Methods:**

We collected ecological momentary assessments (EMAs) via a smartphone app among California young adults (ages 18‐29 y) in 2023‐2024. Participants completed four random prompts each day for 30 consecutive days. Outcomes were defined as whether participants reported being about to vape nicotine, cannabis, or both substances (same-occasion co-vaping) in a given EMA. We used mixed-effects logistic regression models to examine real-time antecedents of each outcome.

**Results:**

Overall, 113 participants (mean age 23.8 y, SD3 y, 63% female, n=70) completed 9001 EMAs. Similar antecedents of all 3 vaping outcomes were craving and using alcohol. Increased cravings for a given substance were associated with a higher likelihood of vaping that substance or co-vaping. Craving for cannabis vaping was associated with lower odds of reporting nicotine vaping (adjusted odds ratio [AOR] 0.87, 95% CI 0.82‐0.92). Feeling happier was associated with higher odds of reporting co-vaping (AOR 1.13, 95% CI 1.01‐1.27) while feeling more stressed was associated with lower odds of vaping nicotine (AOR 0.95, 95% CI 0.91‐0.98) or cannabis (AOR 0.91, 95% CI 0.86‐0.97). Seeing tobacco advertisements was associated with higher odds of vaping nicotine (AOR 3.09, 95% CI 1.48‐6.46) and co-vaping (AOR 4.15, 95% CI 1.18‐14.52). Cannabis vaping was more likely to occur in the afternoon (AOR 1.52, 95% CI 1.16‐1.98) and nighttime (AOR 1.95, 95% CI 1.45‐2.63) than in the morning. Co-vaping was also more likely to occur in the afternoon (AOR 1.59, 95% CI 1.14‐2.22) and nighttime (AOR 1.84, 95% CI 1.26‐2.71) than in the morning, but the association was not held for nicotine vaping. Nicotine vaping was more likely to occur on weekends compared to weekdays (AOR 1.25, 95% CI 1.09‐1.45), but no significant associations were found for cannabis vaping and co-vaping.

**Conclusions:**

We found similar antecedents (craving and alcohol use) and unique antecedents (mood, advertising exposure, and time of day) for nicotine vaping, cannabis vaping, and same-occasion co-vaping, suggesting targets for future vaping cessation interventions.

## Introduction

Using vaporizers to vape nicotine and cannabis (vaping) is common among US young adults (ages 18‐29 y), with 24.1% reporting vaping nicotine and 12.6% reporting vaping cannabis in the past month in 2023 [1]. Co-use of both substances is also common, with 54.6% of young adults who vape nicotine also vaping cannabis [2]. While the long-term effects of vaping are not yet fully understood, evolving evidence has shown that vaping nicotine and cannabis exposes users to toxic chemicals (eg, propylene glycol, aldehydes, and acrolein) that may increase the risk for respiratory and cardiovascular diseases [3-5]. Young adulthood is a critical period for intervention since this developmental period includes transitions from substance use experimentation to established use [6,7]. Of young people who vape nicotine, over half desire to quit [8]. However, vaping cessation interventions for this population are still scarce.

Young adult vaping exhibits complex behavioral patterns. Young adults may engage in nicotine and cannabis vaping by using each substance on separate days within the same month (same-month co-vaping or single-substance vaping), using both substances on the same day (same-day co-vaping), or using both on the same occasion (same-occasion co-vaping) [9]. Closer temporal proximity of co-vaping was associated with greater intensities of vaping nicotine and cannabis [10], with the pattern of same-occasion co-vaping having the highest vaping consumption compared to other patterns. Understanding predictors of different vaping patterns is critical to informing interventions curbing nicotine and cannabis vaping.

Ecological momentary assessment (EMA) has been established as a data collection method to capture fine-grained information on vaping behaviors in daily life [11]. EMA uses mobile devices to repeatedly assess a targeted behavior as it occurs in naturalistic settings. It has been used successfully to investigate antecedents of substance use behaviors while reducing recall bias and improving ecological validity. This data collection method is ideal for examining subjective and contextual factors that trigger nicotine and cannabis vaping in real time, which could not be captured by using traditional data collection methods [12,13]. Several EMA studies have examined different behaviors of using tobacco (eg, exclusive e-cigarette use, exclusive cigarette use, and dual tobacco use) and cannabis among young adults [14-17]. However, it is unclear whether real-time antecedents differ between nicotine and cannabis vaping, and less is known about real-time antecedents of same-occasion co-vaping.

To address this gap, we aimed to examine real-time antecedents of nicotine and cannabis vaping and same-occasion co-vaping among young adults. Such data might inform the development of interventions to reduce nicotine and cannabis vaping and related harms in this population, such as just-in-time adaptive intervention, which delivers personalized and adaptive supports tailored to antecedents at moments when there is a high likelihood of vaping occurrence.

## Methods

### Design and Procedures

We conducted a smartphone-based EMA study on vaping behaviors among young adults. The study was conducted in California during June 2023-January 2024. Details about study design and participant recruitment have been reported elsewhere [10]. Briefly, participants completed an online survey through Qualtrics that asked about their demographics, substance use history, current vaping behaviors, and other characteristics (eg, perceptions of vaping) at baseline. Participants downloaded the study app on their own smartphones and were guided on using the app to collect EMAs for a 30-day period consecutively. Each day, participants were prompted by the study app to complete 4 random surveys during waking hours and were asked to report substance vaping at that specific moment. Each EMA required 2‐3 minutes to complete and was available only for 30 minutes. All responses were time and date-stamped to allow for a time-specific analysis.

### Participants

Participants were recruited through Instagram. The advertisement contained a link to the study’s screener. Eligible participants were 18‐29 years of age, resided in California, owned a smartphone, reported vaping either nicotine or cannabis at least 20 days during the past month, and intended to quit vaping either nicotine or cannabis in the next 6 months. Eligible participants were required to send a picture of their ID to verify their identity. This study followed the Strengthening the Reporting of Observational Studies in Epidemiology (STROBE) guidelines.

### Measures

#### Outcome Variables

We examined 3 binary vaping outcomes, including momentary nicotine vaping, momentary cannabis vaping, and momentary co-vaping of both nicotine and cannabis on the same occasion. These outcomes were derived from participants’ responses to the questions about vaping behaviors in each EMA (eg, “Are you about to vape right now? [yes, no];” If answering “yes,” then being asked “What type of vapes? [nicotine, cannabis]”). In this study, “about to vape” was defined as the moment immediately before initiating vaping, when the participant was physically engaged with or preparing to use a vaping device. EMAs that reported both nicotine and cannabis vaping were categorized as same-occasion co-vaping, while those reporting no vaping or single-substance vaping were classified as no same-occasion co-vaping.

#### Independent Variables

Based on the extant literature on young adult vaping [15,17,18], we developed items capturing vaping antecedents. Each EMA asked about both subjective and contextual factors of the situation the participant was in at the time of the survey. Subjective factors included craving and mood. Nicotine and cannabis cravings were measured by asking participants, “How strong is your urge to vape nicotine/cannabis?” with the response option on a Likert scale from 0 (not at all) to 6 (very high). Participants’ moods were measured by 4 separate questions, including stressed, energized, happy, and focused levels (eg, “Right now, how [happy, stressed, focused, energized] do you feel?”). The response for each item ranged from 0=Not at all to 10=Extremely.

Contextual factors included alcohol consumption (yes or no), the presence of specific triggers (eg, seeing a nicotine or cannabis vaping product or advertisement), time of the day (ie, morning: 5 AM to 12 noon; afternoon: 12 noon to 6 PM; and evening or night: 6 PM to 5 AM), and day of the week (weekend vs weekday). In addition, participants were asked about the presence of other people, and the responses were categorized as alone, with roommate(s) or friend(s), partner, family, and multiple types of people or acquaintances.

#### Baseline Characteristics

Data on demographics included age, biological sex, sexual orientation, race and ethnicity, marital status, and educational attainment. In addition, e-cigarette dependence was measured using the Penn State Electronic Cigarette Dependence Index, and the total dependence score (ranging from 0 to 20) was treated as a continuous variable [19]. Cannabis use disorder was assessed using the short form of the Cannabis Use Disorder Identification Test-Revised (CUDIT-R), with a cutoff score of 13 or greater being categorized as having cannabis use disorder [20].

### Statistical Analysis

Descriptive statistics were summarized for the vaping outcomes, use of other tobacco and cannabis products, and other characteristics. Based on previous EMA studies [21,22] and recommended analytic methods for EMAs [23], we used generalized linear mixed-effects models (GLMMs) to examine real-time antecedents of the vaping outcomes. The GLMMs are well-suited for analyzing EMA data because they can account for repeated measures nested within individuals, allow modeling of time-varying predictors, and incorporate random effects to capture both within- and between-person variability. Separate GLMMs with a logistic link function were used to examine predictors for each of the binary outcomes (ie, nicotine vaping, cannabis vaping, and same-occasion co-vaping of both substances). Models included random effects for subjects and fixed effects for predictors. All variables were included simultaneously in the models. Baseline covariates, including demographic variables, e-cigarette dependence, and cannabis use disorder were also controlled in the models. We conducted sensitivity analyses among participants who had a compliance rate of at least 75% to assess the robustness of the main findings. All tests were 2-tailed with a significance level of α less than .05. Statistical analyses were performed using Stata version 18 (StataCorp).

### Ethical Considerations

The University of California, San Francisco Institutional Review Board approved the study (approval 22-36715). Participants were provided with detailed information about the study, and electronic informed consent was obtained through the study website. To maximize participant compliance with EMAs, incentives were tied to completed EMA surveys, with higher incentives for better compliance, ranging from US $0 to US $120 gift cards. Those who completed more than 80% of surveys received a bonus incentive of US $60. Personal details of participants were kept confidential, and a unique study identification number was assigned to each participant for data entry, management, and analysis.

## Results

### Sample Characteristics

Table 1 shows the baseline characteristics of the 113 participants. The majority were female, single, and had an education attainment of college or higher. Participants had diverse racial and ethnic backgrounds and sexual orientation. The average e-cigarette dependence score was 10 (SD 5), and most participants met the criteria for cannabis use disorder.

**Table 1.** Study participant characteristics at baseline.

Demographic characteristics	Participants (N=113)
Age, mean (SD)	23.8 (3.0)
Sex assigned at birth (female), n (%)	70 (63)
Sexual orientation, n (%)	
Straight	63 (56)
Bisexual	31 (27)
Gay or lesbian	11 (10)
Other	8 (7)
Marital status, n (%)	
Married or living with partner	34 (30)
Never married	79 (70)
Race and ethnicity, n (%)	
Non-Hispanic: Asian	29 (26)
Non-Hispanic: Black	3 (3)
Hispanic	33 (29)
Non-Hispanic: White	35 (31)
Non-Hispanic: others and multiraces	13 (11)
Education, n (%)	
Less than high school	5 (5)
Completed high school	15 (13)
Some college—no degree or associate degree	50 (44)
Completed bachelor’s degree or higher	43 (38)
e-Cigarette dependence index: PS-score^a^, mean (SD)	10.1 (4.8)
Cannabis use disorder, n (%)	89 (79)

a PS-core: Penn State Electronic Cigarette Dependence Index.

### Ecological Momentary Assessments of Vaping Behaviors and Real-Time Factors

A total of 9001 EMAs (out of 13,560 EMAs) were completed by the participants for an average compliance of 66.4% (Table 2). Nicotine vaping was reported in 35.8% (n=3225) of the total assessments, cannabis vaping was reported in 9.3% (n=839), and same-occasion co-vaping of nicotine and cannabis in 6.3% (n=570).

**Table 2.** Distribution of vaping outcomes and real-time predictors reported in ecological momentary assessment (EMA) during the study time.

Real-time variables	All observations (n=9001)	Compliance rate≥75% (n=6563)
Outcomes, n (%)		
Nicotine vaping	3225 (35.8)	2481 (37.8)
Cannabis vaping	839 (9.3)	546 (8.3)
Same-occasion co-vaping (vaping both substances at the same moment)	570 (6.3)	418 (6.4)
Real-time predictors		
Personally staying with, n (%)		
Someone else or multiple people	1466 (16.3)	1125 (17.1)
Alone	4421 (49.1)	3107 (47.3)
Roommate or friend	774 (8.6)	509 (7.8)
Partner	1491 (16.6)	1175 (17.9)
Family	849 (9.4)	647 (9.9)
Product advertisement exposure, n (%)		
Seeing tobacco products	922 (10.2)	580 (8.8)
Seeing tobacco advertisement	129 (1.4)	96 (1.5)
Seeing cannabis products	638 (7.1)	284 (4.3)
Seeing cannabis advertisement	115 (1.3)	73 (1.1)
Alcohol use, n (%)	407 (4.5)	271 (4.1)
Time of the day, n (%)		
Morning (5 AM-12 PM)	2114 (23.5)	1565 (23.9)
Afternoon (12 PM-6 PM)	4584 (50.9)	3356 (51.1)
Evening or night (6 PM-5 AM)	2303 (25.6)	1642 (25)
Weekday (vs weekend), n (%)	6587 (73.2)	4807 (73.2)
Mood (0‐10)^a^, mean (SD)		
Feeling happy	5.9 (2.2)	6.1 (2.1)
Feeling stressed	3.6 (2.6)	3.4 (2.6)
Feeling focused	5.2 (2.2)	5.4 (2.2)
Feeling energized	5.3 (2.2)	5.6 (2.2)
Craving for nicotine (0‐6)^b^, mean (SD)	2.9 (1.9)	3.0 (1.9)
Craving for cannabis (0‐6)^b^, mean (SD)	1.7 (1.9)	1.6 (1.9)

a The scale for each emotional feeling ranged from 0=Not at all to 10=Extremely.

b Craving score ranged from 0=Not at all to 6=Very high.

In almost half of the assessments (49.1%, n=4421) participants were alone, followed by being with a partner (16.6%, n=1491), multiple people or someone else (16.3%, n=1466), family (9.4%, n=849), and roommates or friends (8.6%, n=774). Participants reported seeing tobacco products in 10.2% (n=922) and tobacco advertisements in 1.4% (n=129) of the assessments, while proportions for seeing cannabis products and advertisements were 7.1% (638) and 1.3% (n=115), respectively. Alcohol use was reported in 4.5% (n=407) of the assessments.

Among 65 participants (57.5% of the total sample) who had an EMA compliance rate of 75% or greater, 6563 EMAs were completed (average compliance of 84%), which included nicotine vaping being reported in 2481 assessments (37.8%), cannabis vaping in 546 assessments (8.3%), and same-occasion co-vaping in 418 assessments (6.4%). The distribution of real-time antecedents was slightly different compared to those among the entire sample.

### Real-Time Antecedents of Nicotine Vaping

Nicotine craving was positively associated with the odds of vaping nicotine, whereas craving for cannabis was negatively associated with vaping nicotine (adjusted odds ratio [AOR] 0.87, 95% CI 0.82‐0.92; see Table 3). Feeling stressed (AOR 0.95, 95% CI 0.91‐0.98) and energized (AOR 0.93, 95% CI 0.88‐0.98) were negatively associated with the likelihood of vaping nicotine. Participants were more likely to vape nicotine when being alone (AOR 2.81, 95% CI 2.27‐3.46) or with roommates or friends (AOR 2.06, 95% CI 1.55‐2.72) or a partner (AOR 2.98, 95% CI 2.32‐3.82) compared to being with multiple types of people or acquaintances (see Table 3). Seeing tobacco products (AOR 6.56, 95% CI 5.01‐8.59) and tobacco advertisements (AOR 3.09, 95% CI 1.48‐6.46) increased the odds of vaping nicotine. Alcohol use was associated with higher odds of vaping nicotine (AOR 1.55, 95% CI 1.13‐2.12). Participants tended to vape nicotine more on weekends than on weekdays (AOR 1.25, 95% CI 1.09‐1.45), but the time of the day was not associated with vaping nicotine.

**Table 3.** Factors associated with momentary vaping behaviors among young adults. All models were controlled for study day (1-30) and baseline covariates, including age (years), biological sex (male vs female), sexual orientation (nonstraight vs straight), cannabis use disorder, e-cigarette dependence score, race and ethnicity (Non-Hispanic White, Hispanic, and Non-Hispanic-Other), and education levels.

Real-time factors	All observations (n=8911)		
	Nicotine vaping, AOR^a^ (95% CI)	Cannabis vaping, AOR (95% CI)	Same-occasion co-vaping, AOR (95% CI)
Mood^b^			
Feeling happy	1.02 (0.96‐1.08)	1.07 (0.98‐1.15)	1.13 (1.01‐1.27)^c^
Feeling stressed	0.95 (0.91‐0.98)^d^	0.91 (0.86‐0.97)^d^	0.89 (0.82‐0.97)^d^
Feeling focused	1.03 (0.99‐1.09)	1.00 (0.94‐1.08)	1.01 (0.92‐1.11)
Feeling energized	0.93 (0.88‐0.98)^c^	1.00 (0.93‐1.08)	0.93 (0.84‐1.03)
Craving for nicotine^e^	2.38 (2.25‐2.53)^f^	0.96 (0.87‐1.05)	1.22 (1.07‐1.38)^d^
Craving for cannabis^e^	0.87 (0.82‐0.92)^f^	2.33 (2.15‐2.53)^f^	2.21 (1.98‐2.47)^f^
Personally staying with			
Someone else or multiple people	Ref^g^	Ref	Ref
Alone	2.81 (2.27‐3.46)^f^	2.24 (1.59‐3.16)^f^	1.65 (1.06‐2.56)^c^
Roommate or friend	2.06 (1.55‐2.72)^f^	1.97 (1.24‐3.12)^d^	2.35 (1.28‐4.29)^d^
Partner	2.98 (2.32‐3.82)^f^	2.29 (1.53‐3.44)^f^	1.71 (1.02‐2.86)^c^
Family	0.97 (0.73‐1.31)	1.04 (0.63‐1.72)	1.21 (0.66‐2.23)
Product and advertisement exposure			
Seeing tobacco products	6.56 (5.01‐8.59)^f^	1.50 (1.03‐2.20)^c^	1.72 (1.01‐2.93)^c^
Seeing tobacco advertisement	3.09 (1.48‐6.46)^d^	1.64 (0.62‐4.33)	4.15 (1.18‐14.52)^c^
Seeing cannabis products	0.86 (0.61‐1.21)	3.68 (2.58‐5.25)^f^	3.90 (2.29‐6.68)^f^
Seeing cannabis advertisement	0.97 (0.45‐2.11)	0.81 (0.37‐1.77)	0.72 (0.27‐1.93)
Alcohol use (Yes vs No)	1.55 (1.13‐2.12)^d^	1.87 (1.24‐2.80)^d^	2.25 (1.40‐3.62)
Time of day			
Morning (5 AM-12 PM)	Ref	Ref	Ref
Afternoon (12 PM-6 PM)	1.04 (0.89‐1.22)	1.52 (1.16‐1.98)^d^	1.59 (1.14‐2.22)^d^
Evening or night (6 PM-5 AM)	1.16 (0.96‐1.40)	1.95 (1.45‐2.63)^f^	1.84 (1.26‐2.71)^d^
Weekend vs weekday	1.25 (1.09‐1.45)^d^	0.92 (0.74‐1.15)	0.86 (0.65‐1.13)

a AOR: adjusted odds ratio.

b The scale for each emotional feeling ranged from 0=Not at all to 10=Extremely.

c *P*<.05.

d *P*<.01.

e Craving score ranged from 0=Not at all to 6=Very high.

f *P*<.001.

g Ref: Reference.

### Real-Time Antecedents of Cannabis Vaping

Craving for cannabis was a significant antecedent (AOR 2.33, 95% CI 2.15‐2.53) of cannabis vaping. Regarding mood, participants were less likely to vape cannabis as they felt more stressed (AOR 0.91, 95% CI 0.86‐0.97). Participants were more likely to report cannabis vaping when they were alone (AOR 2.24, 95% CI 1.59‐3.16), with a roommate(s) or friend(s) (AOR 1.97, 95% CI 1.24‐3.12) or a partner (AOR 2.29, 95% CI 1.53‐3.44) compared to being with other people. Seeing tobacco products (AOR 1.50, 95% CI 1.03‐2.20), seeing cannabis products (AOR 3.68, 95% CI 2.58‐5.25), and alcohol use (AOR 1.87, 95% CI 1.24‐2.80) were also significant antecedents of cannabis vaping. Participants were more likely to vape cannabis in the afternoon (AOR 1.52, 95% CI 1.16‐1.98) and in the evening (AOR 1.95, 95% CI 1.45‐2.63) compared to the morning, but there was no statistically significant difference between weekdays and weekends.

### Real-Time Antecedents of Same-Occasion Co-Vaping of Nicotine and Cannabis

Both cravings for nicotine (AOR 1.22, 95% CI 1.07‐1.38) and cannabis (AOR 2.21, 95% CI 1.98‐2.47) were associated with higher increased odds of co-vaping. The higher levels of feeling happy were positively associated with co-vaping (AOR 1.13, 95% CI 1.01‐1.27), while higher levels of feeling stress were negatively associated with co-vaping. Similar to nicotine vaping and cannabis vaping, participants were more likely to co-vape when they were alone, with roommates or friends, or with a partner compared to being with other people. Likewise, seeing tobacco products, seeing tobacco advertisements, seeing cannabis products, and alcohol use were other real-time antecedents of co-vaping. Similar to cannabis vaping, participants were also more likely to co-vape both substances in the afternoon or evening compared to the morning time, but there were no statistical differences in the likelihood of co-vaping between weekends and weekdays.

### Sensitivity Analyses

Results of the sensitivity analyses with the subset of 65 participants (57.5% of the total sample) who had a compliance rate of 75% or greater are shown in Table 4. The rates were mostly the same for vaping nicotine and vaping cannabis outcomes. Some associations turned nonsignificant in the model for co-vaping, but the direction of most associations remained the same with results from analyzing observations from the entire sample.

**Table 4.** Sensitivity analyses of factors associated with momentary vaping behaviors among young adults completing at least 75% of ecological momentary assessment (EMA) surveys during the study period. All models were controlled for study day (1-30) and baseline covariates, including age (years), biological sex (male vs female), sexual orientation (non-straight vs straight), cannabis use disorder, e-cigarette dependence score, race and ethnicity (Non-Hispanic White, Hispanic, and Non-Hispanic–Other), and education levels.

Real-time factors	Compliance rate ≥75% (n=6473)		
	Nicotine vaping, AOR^a^ (95% CI)	Cannabis vaping, AOR (95% CI)	Same-occasion co-vaping, AOR (95% CI)
Mood^b^			
Feeling happy	0.97 (0.91‐1.04)	1.11 (0.99‐1.24)	1.16 (1.01‐1.35)^c^
Feeling stressed	0.92 (0.87‐0.96)^d^	0.88 (0.81‐0.96)^e^	0.91 (0.82‐1.01)
Feeling focused	1.05 (0.99‐1.11)	1.06 (0.97‐1.16)	1.07 (0.96‐1.20)
Feeling energized	0.91 (0.86‐0.98)^e^	1.01 (0.92‐1.11)	0.97 (0.86‐1.09)
Craving for nicotine^f^	2.48 (2.30‐2.66)^d^	0.87 (0.77‐0.98)^c^	1.11 (0.95‐1.30)
Craving for cannabis^f^	0.87 (0.81‐0.93)^d^	2.53 (2.27‐2.82)^d^	2.35 (2.04‐2.71)^d^
Personally staying with			
Someone else or multiple people	Ref^g^	Ref	Ref
Alone	2.67 (2.09‐3.41)^d^	1.58 (1.02‐2.45)^c^	1.13 (0.65‐1.96)
Roommate or friend	2.01 (1.43‐2.81)^d^	1.87 (1.02‐3.42)^c^	2.13 (0.96‐4.76)
Partner	3.35 (2.51‐4.46)^d^	2.21 (1.34‐3.64)^e^	1.59 (0.85‐2.97)
Family	1.05 (0.75‐1.48)	0.79 (0.42‐1.45)	0.86 (0.41‐1.80)
Product and advertisement exposure			
Seeing tobacco products	6.76 (4.88‐9.37)^d^	2.06 (1.17‐3.61)^c^	2.34 (1.04‐5.26)^c^
Seeing tobacco advertisement	4.16 (1.47‐11.75)^e^	1.76 (0.41‐7.50)	3.08 (0.39‐24.06)
Seeing cannabis products	1.00 (0.61‐1.66)	3.10 (1.77‐5.44)^d^	4.68 (1.93‐11.34)^e^
Seeing cannabis advertisement	0.28 (0.07‐1.66)	0.59 (0.12‐2.82)	0.86 (0.10‐7.46)
Alcohol use	1.37 (0.93‐2.04)	1.42 (0.83‐2.42)	1.64 (0.89‐3.02)
Time of day			
Morning (5 AM-12 PM)	Ref	Ref	Ref
Afternoon (12 PM-6 PM)	1.06 (0.88‐1.28)	1.42 (1.02‐1.98)^c^	1.38 (0.93‐2.07)
Evening or night (6 PM-5 AM)	1.22 (0.97‐1.53)	1.84 (1.26‐2.70)^e^	1.75 (1.10‐2.78)^c^
Weekend vs weekday	1.37 (1.15‐1.62)^d^	0.99 (0.74‐1.31)	0.97 (0.69‐1.35)

a AOR: adjusted odds ratio.

b The scale for each emotional feeling ranged from 0=Not at all to 10=Extremely.

c *P*<.05.

d *P*<.001.

e *P*<.01.

f Craving score ranged from 0=Not at all to 6=Very high.

g Ref: Reference.

## Discussion

### Principal Findings

This smartphone-based EMA study revealed real-time antecedents of vaping behaviors, including nicotine vaping, cannabis vaping, and especially same-occasion co-vaping. To the best of our knowledge, this is the first study that examines the antecedents of same-occasion co-vaping of nicotine and cannabis among young adults, a high-risk behavioral pattern for vaping dependence [10]. Our study identified both common and unique antecedents across the vaping behavioral patterns (eg, craving and alcohol use were shared predictors for both nicotine and cannabis vaping behaviors while feeling happy was a unique antecedent for same-occasion co-vaping). The findings suggest there may be different targets for cessation programs to help young adults quit vaping nicotine, cannabis, or same-occasion co-vaping.

### Interpretation of Results and Comparison With Previous Work

We found that higher craving levels of a given substance were not only associated with an increased likelihood of vaping that substance but also co-vaping. Young adults may vape both substances on the same occasion to seek combined pharmacological effects from the combination of substances [9,24]. The co-vaping behavior may increase the amount of vaping nicotine and cannabis, putting young adults at an increased risk for nicotine and cannabis dependence [10]. On the other hand, we found that craving for cannabis was associated with lower odds of vaping nicotine, while craving for nicotine was not associated with vaping cannabis. This finding does not support substitution effects between nicotine and cannabis (eg, using one product to replace or substitute the other [25]). A behavioral economic study among adults who use both cannabis and tobacco also found that cannabis joints and cigarettes had an independent relationship [26]. These mixed findings about substitution effects are probably due to cannabis and tobacco having both overlap and unique salient purposes of use [27]. Although cannabis use can trigger or increase cravings for tobacco, and vice versa, our participants were motivated to quit and might have intentionally avoided using one substance to prevent triggering cravings for the other. Cannabis and nicotine vaping share some overlapping motives (eg, stress relief and relaxation) but also serve distinct purposes. For example, nicotine vaping may be used as a substitute for combustible tobacco, while cannabis vaping may be used for sleep or medical reasons. These shared and unique motivations may help explain both independent and substitution patterns in tobacco and cannabis use. Future studies should consider the underlying purposes of each substance use when examining co-use behaviors.

Our participants were less likely to vape nicotine and cannabis as they had higher levels of stress, which contrasts with previous studies indicating stress as a common reason for nicotine vaping among young adults [28-32]. However, previous studies measured vaping and stress within 30 days and examined the between-person association between stress and vaping [28,29,32], while our study measured stress levels and the vaping outcomes at a specific moment and examined this association as a within-person variation. Thus, differences in the measurement of exposures and outcomes could be attributed to the difference between our findings and other studies’ findings. Some studies even indicate that nicotine vaping can increase anxiety symptoms and stress levels [33,34], whereas the self-medication hypothesis suggested that people may vape to reduce stress and depression symptoms [35,36]. Therefore, how young adults perceive the roles of each substance on their mental health and mood could alter this association. Feeling happy, in contrast, was positively associated with the likelihood of co-vaping among young adults in our study. When participants feel happy, they may reward themselves by vaping both nicotine and cannabis on the same occasion to “get the buzz” of the effect of cannabis to enhance their positive mood [25,37]. Future studies should further examine mood variation and vaping behaviors, adjusting for perceptions of the roles of substance use on mental health to better understand potential mechanisms underlying the influence of mood and vaping.

We also found that alcohol use was significantly associated with all the vaping outcomes, which is consistent with previous studies [38,39]. Alcohol, tobacco, and cannabis can potentiate rewarding properties of each other [24,40,41], which may increase the use and dependence on each substance. Thus, a single substance use cessation program may not work effectively without considering the concurrent use of multiple substances. In addition, a previous study indicated that vaping nicotine and cannabis occurs more in social events (eg, attending bars or parties) [38], but we found that vaping nicotine, cannabis, and co-vaping consistently occurred more frequently not only when young adults were staying with their roommates or friends, or a partner, but also when they were alone compared to when staying with multiple types of people or acquaintances. This finding underscores the dynamic nature of vaping behaviors depending on context and social setting.

Seeing tobacco products and advertisements was associated with higher odds of vaping nicotine and same-occasion co-vaping, which is consistent with previous studies [42,43]. Likewise, seeing cannabis products was also associated with cannabis vaping and co-vaping. This finding highlights the need to address exposures to nicotine and cannabis products and advertisements and guide young adults to avoid these triggers to accommodate their vaping cessation goals.

In our study, time of the day was not associated with vaping nicotine, but cannabis vaping and co-vaping were more likely to occur in the afternoon or evening. Young adults may be chained to vaping nicotine as a habit while vaping cannabis for certain purposes (eg, sleep aid or pain management) [44,45], which may partly explain the observed differences in the timing of their vaping behaviors. In addition, while nicotine vaping was associated with weekends (vs weekdays), neither cannabis nor co-vaping was associated with weekends. These findings have implications for delivering cessation interventions at the right times to tailor content and timing of messages targeting each vaping behavior. Just-in-time adaptive interventions are grounded in the idea that timing of states of vulnerability and opportunity plays a critical role in determining the most beneficial moments to deliver support [46]. This type of intervention has been applied for smoking cessation and could offer a promising approach to deliver vaping cessation interventions via smartphone apps [47,48]. For example, as we found that cannabis vaping and co-vaping were more likely to occur in the afternoon and evening, future studies targeting these behavioral outcomes may consider sending push notifications or intervention messages during these times of the day. In addition, combining data from EMAs and mobile sensing or wearable sensors could help to improve our understanding of vaping behaviors and their real-time triggers and inform the development of just-in-time adaptive interventions [11]. For example, EMA and sensor data can be combined to train a machine learning algorithm to detect high-risk moments before nicotine and cannabis vaping. When sensor data (eg, motion, temperature, and heart rate) suggest craving or use, a smartphone app can trigger an EMA to assess context and deliver a tailored message.

### Limitations

Limitations to this study include a convenience sample and online recruitment of young adults in California. Substantial proportions of our sample had educational attainment of college or higher, cannabis use disorders, and high e-cigarette dependence. In addition, our participants were interested in quitting vaping, so they might be more conscious of their vaping behaviors or respond differently to vaping triggers compared with those not considering quitting. Thus, it is unclear how our findings can be generalized to other samples of young adults. As most cessation interventions target individuals who are motivated to quit, our findings may provide critical implications for supporting young adults in the contemplation stage or later stages of behavior change [49]. There is a possibility of social desirability bias as participants may underreport their vaping behaviors. Finally, attentional lapses may affect the accuracy of participants’ responses, particularly in the context of frequent momentary assessments.

### Conclusions

In conclusion, this EMA study revealed how subjective and contextual factors differentially predicted nicotine vaping, cannabis vaping, and same-occasion co-vaping among young adults. In addition to similar antecedents for vaping behaviors, we also found unique antecedents for each vaping outcome, informing the development of just-in-time adaptive interventions to help young adults quit vaping nicotine and cannabis. As the overlap between nicotine and cannabis vaping is increasing, future research should address co-vaping of both substances and develop dual cessation treatment among young populations.

## Abbreviations

AOR: adjusted odds ratio
CUDIT-R: Cannabis Use Disorder Identification Test-Revised
EMA: ecological momentary assessment
